# Influences of NaCl and Na_2_SO_4_ on the Micellization Behavior of the Mixture of Cetylpyridinium Chloride + Polyvinyl Pyrrolidone at Several Temperatures

**DOI:** 10.3390/gels8010062

**Published:** 2022-01-16

**Authors:** Md. Farid Ahmed, Malik Abdul Rub, Md. Tuhinur R. Joy, Mohammad Robel Molla, Naved Azum, Md. Anamul Hoque

**Affiliations:** 1 Department of Chemistry, Jahangirnagar University, Savar, Dhaka 1342, Bangladesh; ahmedfarid844@gmail.com (M.F.A.); musayebju40@gmail.com (M.R.M.); ahoque_ju@yahoo.com (M.A.H.); 2 Bangladesh Council of Scientific and Industrial Research (BCSIR), Dhaka 1205, Bangladesh; 3 Chemistry Department, Faculty of Science, King Abdulaziz University, Jeddah 21589, Saudi Arabia; nhassan2@kau.edu.sa; 4 Center of Excellence for Advanced Materials Research, King Abdulaziz University, Jeddah 21589, Saudi Arabia; 5 Department of Chemistry, Jashore University of Science and Technology, Jashore 7408, Bangladesh; joytuhinur@yahoo.com

**Keywords:** polyvinyl pyrrolidone, critical micelle concentration, cetylpyridinium chloride, thermodynamics, conductivity measurement

## Abstract

Herein, the conductivity measurement technique is used to determine the interactions that may occur between polyvinyl pyrrolidone (PVP) polymer and cetylpyridinium chloride (CPC) surfactant in the presence of NaCl and Na_2_SO_4_ of fixed concentration at variable temperatures (298.15–323.15 K) with an interval of 5 K. In the absence or presence of salts, we observed three critical micelle concentrations (*CMC*) for the CPC + PVP mixture. In all situations, *CMC*_1_ values of CPC + PVP system were found to be higher in water than in attendance of salts (NaCl and Na_2_SO_4_). Temperature and additives have the tendency to affect counterion binding values. Various physico-chemical parameters were analyzed and demonstrated smoothly, including free energy (Δ*G*^0^_m_), enthalpy (Δ*H*^0^_m_) and entropy change (Δ*S*^0^_m_). The micellization process is achieved to be spontaneous based on the obtained negative Δ*G*^0^_m_ values. The linearity of the $\Delta H_{m}^{o}$ and $\Delta S_{m}^{o}$ values is excellent. The intrinsic enthalpy gain (Δ*H*^0*^_m_) and compensation temperature (*T*_c_) were calculated and discussed with logical points. Interactions of polymer hydrophobic chains or the polymer + surfactant associated with amphiphilic surface-active drugs can employ a strong impact on the behavior of the gels.

## 1. Introduction

Surfactants are substances that can reduce the surface tension of a solvent. Aggregation behavior of amphiphilic substances, which is basically a non-covalent interaction, is a normal and spontaneous phenomenon [1]. The structural configuration of surfactant molecules, and the presence of polar and non-polar portions, helps them to be self-assembled in an aqueous medium at a certain concentration which is termed as critical micelle concentration (*CMC*) [2]. Micelles can easily incorporate feebly soluble drugs, organic chemicals, or polymers inside their hydrophobic core and facilitate the solubility of various substances and bioavailability as well [3,4]. However, surfactants act as a good recipient regarding drug delivery systems. Studies have suggested that insertion of external substances may impact on the physical properties of aggregates such as adding or departure of counter ion binding, reaction rate, and catalytic activity [5,6]. Gelation is the gel preparation process from a system through polymers. Functions of gels are established by their drug-loading ability, rheological behavior, and the mechanisms and kinetics of drug discharge. The integration of small ratios of surfactants, which can elevate or impede intra/interchain polymeric bonds, can change these assets and be a valuable tool for emerging gel-based dosage forms [7,8].

It is inevitable that this aggregate nature of surfactant molecules has made it a perfect candidate for its versatile potential applications. In the case of pharmaceutical research, cosmetics and perfumes industries, antiseptics, and disinfectants, surfactants are used due to their good chemical and anti-microbial properties [5]. Surfactants can be utilized to prepare antibacterial hydrogels. Shunji Yunoki et al. [9] studied that cetylpyridinium chloride (CPC) and polyvinyl alcohol (PVA) based antibacterial hydrogels showed excellent antibacterial properties where interaction between the used surfactant and polymer was significant. Dye-surfactant interaction for formulation in textile and various coloring industries deserves a remarkable role [10]. Recent research revealed that rinsing the oral captivity can restrain and counteract the menace of transmission of SARS-CoV-2 [11,12]. A recent publication anticipated that CPC has capability to destroy viral capsids [13] and can become active against different enveloped viruses such as coronaviruses [14]. CPC is a quaternary ammonium salt and cationic surfactant and its structure made it a perfect component for numerous applications for humans such as dental plaque and gingivitis can be reduced by using 0.05% CPC [15]. Mukherjee et al. [16] and Popkin et al. [17] found that CPC exhibits potent, effective, and rapid antiviral response towards influenza and can decrease the duration and severity of the disease.

Investigation of interaction amid surfactants and polymeric substances is very important because these substances are used in many industries such as lubricants, food, sanitizations, detergents, coatings, paints, adhesives, and cosmetics. In a mixture of polymer and surfactant, they individually provide instinct properties such as rheological and interfacial properties, respectively [18]. Polymer and surfactant interaction is important to reduce interfacial areas between non-polar polymeric segments and water when they are linked with non-polar moieties of surfactants to create micelles [19].

**Scheme 1:**
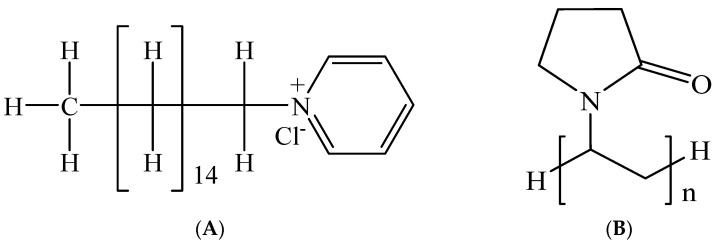
Molecular structure of (**A**) CPC and (**B**) PVP.

Our previously published paper [20] and some other publications have delineated [21,22] the interactions between CPC and polymers/biopolymers in an aqueous medium, but examination of interaction of CPC (Scheme 1A) and polyvinyl pyrrolidone (PVP) (Scheme 1B) in presence of salts at various temperatures, such as in this present work, is rarely studied. Sodium chloride (NaCl), generally known as salt, is a necessary component of our bodies ability to absorb and transport nutrients, regulate blood pressure, and maintain fluid balance. On the other hand, an electrolyte replenisher, sodium sulfate in anhydrous form is used in isosmotic solutions to ensure that delivery does not disrupt normal electrolyte balance or cause water and ion absorption or excretion. Due to the greatest role of these two sodium salts to our body, we have selected these salts. Therefore, in this current work conductivity technique has been used to examine the interaction between the chosen surfactant (CPC) and polymer (PVP) with/without presence of salts (NaCl and Na_2_SO_4_). On the other hand, three different values of *CMC*, fraction of counter ion binding (*β*), thermodynamic parameters such as $\Delta G_{m}^{0},$ $\Delta H_{m}^{0},\;\;\Delta S_{m}^{0}\;\mathrm{and}\;\Delta C_{m}^{0}$ for the aggregation behavior of CPC + PVP mixture in water, and electrolyte solutions, are calculated, expressed, and analyzed thoroughly.

## 2. Experimental

### 2.1. Materials

The materials used in this study were of analytical grade and used without further purification. The chemical names, purity, CAS numbers, mass fraction, and other information are mentioned in Table 1.

### 2.2. Solution Preparation and Conductivity Measurement

CPC + PVP solutions, both in water and salts media, were prepared using distilled-deionized water considering molal concentrations. The specific conductivity of used distilled-deionized water was 1.7–2.0 μS cm^−1^ maintaining temperature range 298.15–323.15 K. Electric balance machine (Mettler Toledo, Greifensee, Switzerland) and 4510 conductivity meter (Jenway, Staffordshire, UK) were used for taking the weight of samples and measuring specific conductivity (*κ*) for preparing different solutions. The conductivity meter had a glass cell electrode specifying cell constant 0.97 cm^−1^ and to calibrate the meter, appropriate concentration of freshly collected KCl solution was employed. Throughout the whole work of conductivity measurement, alternate current (AC) having a frequency of 60 Hz was maintained. In terms of explaining the procedure, initially, 25 mmol kg^−1^ aqueous solution of CPC in PVP was prepared and subsequently, this solution was inserted into the 20 mL solution of PVP at specific temperature both in attendance/non-attendance of salts. Then, salts solutions were also prepared and added to observe the impacts of salts during conductivity study. After every addition of solution, time was maintained to achieve temperature equilibration and conductivity value was recorded; then, this process was employed for every system. Our process of conductivity technique has good matching with others [6,23,24,25]. The RM6 Lauda circulating water bath was used and the error of temperature within ±0.2 K was considered. The values of *CMC* were calculated from the intersection points of *κ* versus concentration of CPC plots for the CPC + PVP assembly by using Origin software.

## 3. Results and Discussion

### 3.1. The CMC and β for the Aggregation of CPC + PVP Mixture in Aqueous and Salts Media

**Figure 1:**
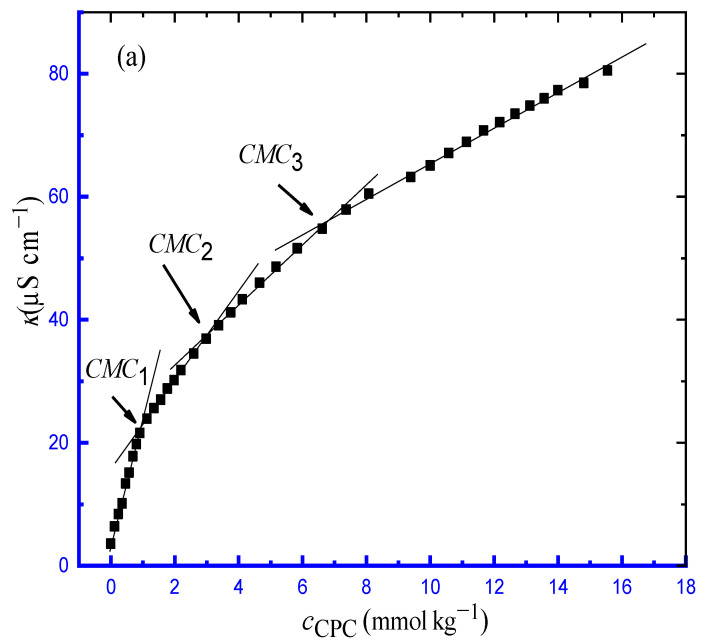

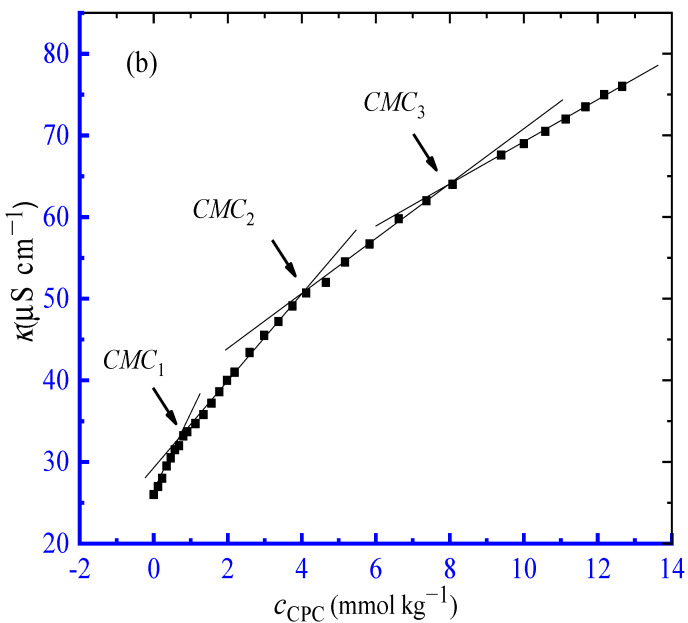
Specific conductivity (*κ*) versus CPC concentration plot for the CPC + 0.1% (*w*/*v*) PVP mixture in (**a**) water and (**b**) aq. 1.50 mmol kg^−1^ NaCl solution at 303.15 K.

The aggregation of surfactants has been investigated by different distinguished research teams applying a number of experimental techniques such as surface tension, conductivity, density, viscosity and ultrasound velocity measurements, fluorescence spectroscopy, NMR Spectroscopy, etc. [26,27]. Among these techniques, conductivity technique is a simple, trustworthy, and broadly used method to evaluate *CMC* for ionic amphiphiles. Ionic surfactants ionize into ions in H_2_O; consequently, specific conductivity has the tendency to increase with enhancing of surfactant contents. Nevertheless, the incremental increase in conductivity undergoes deviation from the initial trend when a certain concentration of surfactant is developed. Such behavior was detected by many researchers in their studies which is mainly owing to the micelle creation [6,20,28,29,30]. The dependence of the specific conductivity, *κ*, on surfactant concentration is shown in Figure 1.

For all the cases of CPC + PVP mixed system, three break points were obtained. The concentration of surfactant achieved at the break point has been taken as the *CMC*, and the successive *CMC* values were expressed as *CMC*_1_, *CMC*_2_, and *CMC*_3_, respectively. The *CMC*_1_ can be associated with the formation of the PVP (polymer): CPC complex (critical aggregation concentration); the *CMC*_2_ corresponds to free CPC micellization (critical micelle concentration in the presence of PVP), and the third break point (*CMC*_3_) refers to the structural modifications in micelles as a sphere to rod transition [31]. The third critical micelle concentration indicates that the spherical micelle turns into a rod shape. Chakraborty et al. [32] described three *CMC*s (critical aggregation concentration (*CAC*), polymer saturation concentration (*c*_s_), and free micellization concentration (*c*_m_^*^)) for the assembly of the mixture of SCMC and CTAB. Bhattarai has achieved three *CMC* values for the aggregation of the CTAB + sodium polystyrene sulfonate mixture [33]. In spite of the existence of multiple *CMC* for an association of polymer + surfactant mixture, a single *CMC* is also stated in the literature [34,35,36,37]. Chai et al. [38] investigated the interaction amid PVP and a gemini surfactant by NMR in a D_2_O medium at 298 K. They confirmed the *CMC*, *СAC*, and additive saturated concentration (*C*_2_) by measuring chemical shift and self-diffusion coefficients, respectively. Mukhim and Ismail [39] reported *CMC* values of 0.841 and 0.75 mmol kg^−1^ for the micellization of CPC in water and 0.32 mmol kg^−1^ NaCl solution, respectively, at 298 K by means of surface tension measurement technique. The decrease in *CMC* values in NaCl solution compared to water medium has good agreement with the current study. Varade et al. [40] investigated the impact of electrolyte (NaCl and NaBr) on the *CMC* value of CPC using different techniques (surface tension/conductance/viscosity/dynamic light scattering (DLS)/small angle neutron scattering). From surface tension and conductivity techniques, Varade et al. [40] stated that CMC value of CPC was found to be 0.98 and 0.95 mM, respectively at 303 K and obtained a decrease in *CMC* value in the occurrence of salt. The DLS study showed that, in presence of electrolytes, the repulsive interactions will cause a rise in the diffusion coefficient and therefore a reduction in the apparent diameter of the micelles, i.e., a decrease in *CMC* of CPC was observed [40].

The extent of micelles dissociation, *α*, has been computed from the ratio of the slopes corresponding to the linear regions below and above *CMC*. If S_1_ and S_2_ are the slopes below and above *CMC*_1_, respectively, then S_2_ and S_3_ are the slopes below and above *CMC*_2_, and S_3_ and S_4_ are the slopes below and above *CMC*_3_, respectively. Then, *α*_1_, *α*_2_, and *α*_3_ can be determined from the ratios S_2_/S_1_, S_3_/S_1_, and S_4_/S_1_, respectively. The fraction of bound counter ions, *β*, at *CMC* can be obtained by subtracting the α value from unity, i.e., *β =* (1 − *α*).

The effect of PVP on the CPC aggregation has been investigated considering the five different concentrations of PVP in the range 0.01–0.10% (*w*/*v*). The *CMC* values for the CPC + PVP system in H_2_O having several concentrations of PVP at 303.15 K are depicted in Table 2. The *CMC* values initially tend to upsurge with the increase in PVP content, attain optimum value, and then undergo decline with the increase in PVP content. Sardar et al. [41] investigated the interactions between PVP and cationic surfactant (both conventional/gemini) while they achieved the enhancement of *CAC* and *CMC* values with the rise of PVP contents [40]. The change of *CMC* values for the CPC + PVP aggregation as a function of PVP content indicates the survival of interaction between CPC and PVP. Additionally, the micelle development phenomenon is a delayed process in the manifestation of PVP.

All the *CMC* values for the CPC + PVP aggregation in aq. NaCl and Na_2_SO_4_ solution have been obtained to be lower in magnitudes in comparison to aqueous medium (Table 3). The reduction in *CMC*s is due to the decreased electrostatic repulsions between the charged head group of the amphiphiles [39]. The effect is much more pronounced in aq. Na_2_SO_4_ solution than in case of aq. NaCl solution. Sulfate ion is multicharged and exists left in the Hoffmeister series compared to the single charged anion Cl^−^. Therefore, the salting out tendency might be more pronounced in case of sulfate which reduces *CMC* to a greater extent at the identical ionic strength [42]. Barbosa et al. [43] achieved three *CMC* values for the SDS +PEO mixture in aq. salts solution including NaCl and Na_2_SO_4_. They also obtained a decreasing trend of *CMC* in the manifestation of these salts. They described that, although NaCl could not interact with PEO, the counter ions of surfactant interact with micelles and macromolecules, which allows the increase in the surfactant’s chemical the potential, and thus the effect of NaCl results in the reduction in both *CAC* and *CMC* values. Akhlaghi and Riahi [44] reported the effect of different salts on the *CMC* of TX-100, and they obtained greater effect of NaCl in reducing *CMC* in comparison to Na_2_SO_4_.

### 3.2. Effects of Temperature on the Association of CPC and PVP Mixture

As the surfactants are used broadly in the applied purposes, its aggregation process experiences an alteration of temperature depending on the seasonal time and applied regions. To understand the impacts of temperature on the aggregation of CPC + PVP mixture, we have selected a range of temperature 298.15–323.15 K in the current investigation, which also covers both room temperature and body temperature. The conductivity and *CMC* values of the CPC + PVP mixture experience a dependency on the temperature variation. The *CMC* values of the CPC + 1% (*w*/*v*) PVP mixture in H_2_O, H_2_O + NaCl, and H_2_O + Na_2_SO_4_ media at several temperatures are shown in Table 3.

In aqueous medium, the *CMC*_1_ values for the aggregation of CPC + PVP mixture undergo an enhancement with the rise of temperature while the *CMC*_2_ and *CMC*_3_ values primarily experience an upsurge with the escalation of temperature, reach an optimum value, and then undergo reduction with the gradual growth of temperature. For the aggregation of CPC + PVP mixture in H_2_O + NaCl medium, the *CMC*_2_ values suffer a fall with the rise of temperature while the *CMC*_1_ and *CMC*_3_ values primarily experience a reduction with the increase in temperature, reach the least value, and then experience an increase with the gradual intensification of temperature. In H_2_O + Na_2_SO_4_ medium, the *CMC*_2_ values undergo a fall with the rise of temperature, while the *CMC*_1_ and *CMC*_3_ values primarily experience a reduction with an increase in temperature, touch the lowest value, and then experience a rise with the gradual intensification of temperature.

### 3.3. Energetics of the Aggregation of CPC + PVP Mixture in Aqueous and Salts Media

The feasibility of the aggregation process can be understood from the knowledge of standard free energy change (Δ*G*^0^*_m_*). It also signifies the spontaneity of the corresponding phenomena. The values of $\Delta G_{m}^{0}$ for CPC + PVP mixture in water and aq. salts solution have been assessed using the following equation [45,46,47,48,49,50,51].

(1) $$\Delta G_{m}^{o}=\left(1+\beta \right)RTlnX_{CMC}$$

The symbols *R*, *T*, and $X_{cmc}\;$in the above equation imply the universal gas constant, study temperature (in Kelvin), and mole fractional value of *CMC*, respectively. The values of $X_{cmc}$ were computed from the ratios of the number of moles of amphiphiles at *CMC* and the overall number of moles existing in the CPC + PVP mixture in H_2_O/aq. salts solutions. The number of moles of H_2_O was considered equal to the ratio of one kilogram and mol. wt. of H_2_O.

The extent of free energy of transfer ($\Delta G_{m,t}^{o}$) for CPC + PVP mixture to shift from H_2_O to H_2_O + salts media was evaluated applying the subsequent Equation (2) [45,52,53]:

(2) $$\Delta G_{m,t}^{0}=\Delta G_{m}^{0}\;\left(H_{2}O+salts\right)-\Delta G_{m}^{0}\;\left(H_{2}O\right)$$

The changes in free energy (Δ*G*^0^*_m_*) values were determined only considering the *CMC*_1_. The values of Δ*G*^0^_1,*m*_ are depicted in Table 4. The Δ*G^0^*_1,m_ values are negative in H_2_O and aq. salts solution. In all the studied media, the negative Δ*G*^0^*_m_* values increase with the growth of experimental temperature. The negative Δ*G*^0^*_m_* values slightly increase in presence of NaCl while the values experience a fall in aq. Na_2_SO_4_ solution. As can be observed, the Δ*G*^0^_1,*m*_ values are negative, indicating that micelles have formed spontaneously in the study solutions. Additionally, the enhanced negative Δ*G*^0^_1,*m*_ values in aq. NaCl solution refer to the increase in the spontaneity for the micelle formation tendency.

This is a common occurrence for the aggregation of surface active materials [25,51]. A similar pattern was reported by Masalci [54], where the Δ*G^0^* values follow the trend we noticed. It is also reported by Masalci [54] that Δ*G*^0^ is shown to fall to higher negative values as the temperature rises when the polymer is increased in quantity. In the absence of electrolytes in the solution, Δ*G^0^*_1,m_ values become more negative as the temperature rises, eventually remaining nearly constant. The drops of Δ*G^0^*_1,m_ values as the temperature rises indicate that the surfactant’s hydrophilic group has desolvated [55]. Sharma et al. [56] reported Δ*G^0^*_m_ value of −16.98 kJ mol^−1^ for the assembly of 0.1% (*w*/*v*) PEG-4000 and CPC mixture, and the Δ*G^0^*_m_ experienced an upsurge with the escalation of temperature. The Δ*G^0^*_m_ value of −29.03 kJ mol^−1^ for the aggregation of PVP + cationic gemini (16-5-16) surfactant at 303 K was stated by Azum et al. [57] and the negative values of their investigation were increased with enhancing temperature. A decrease in spontaneity for the aggregation of the mixture of bovine serum albumin and CPC in aq. glycerol and dimethyl sulfoxide solutions has been achieved by Sharma et al. [58]. In the current study, the free energy of micellization for *CMC*_2_ and *CMC*_3_ were also negative, which revealed the spontaneous occurrence of the processes (not given in tabular form).

The Δ*G^0^*_t_ values are negative in the present investigation for the shift of CPC + PVP system from H_2_O to H_2_O + NaCl medium. The −Δ*G^0^*_t_ values were also obtained for the mixture of TTAB and promethazine hydrochloride in H_2_O + NaCl medium [59]. Similar characteristics of PVP + CPC system on the basis of the values of Δ*G^0^*_t_ have been published for the SCAP + PVP and SDS + PVP system [19,60]. For the aggregation of CPC in aq. 0.05 to 0.5% PVP solutions, Sood obtained Δ*G^0^*_t_ values of −0.49 to –0.64 kJ mol^−1^ while the −Δ*G^0^*_t_ values enhanced slightly with the growing concentration of PVP [19]. The average value of Δ*G^0^*_t_ for CPC + PVP (mol. wt. 40,000) for variation of C_PVP_ from 0.05 to 0.5% and CPC+ PVP (mol. wt. 3, 60,000) for variation of *C*_PVP_ range 0.01 to 0.07% is comparable with our observed data [31]. Azum et al. [53] obtained negative transfer free energy for the micellization of PVP + cationic gemini surfactant, and they obtained no specific trend with temperature. The −Δ*G^0^*_t_ values disclose the feasibility of the interactions between the components present in the system.

Anand and Yadav [61] achieved the negative values of −Δ*G*^0^_*t*_, and the negative values increased and underwent a fall with the enhancing of PVP contents and temperature of the study, respectively. In the case of sodium sulfate solutions, the Δ*G*^0^_*t*_ values are positive, which indicates that the surfactant system prefers to stay in the aqueous medium than the salt solution.

The enthalpy ($\Delta H_{m}^{o}$) and entropy ($\Delta S_{m}^{o}$) changes involved in the aggregation of CPC + PVP mixture were determined using the following equations [45,46,47,48,49,50,51].

(3) $$\Delta H_{m}^{o}=-\left(1+\beta \right)RT^{2}\left(\frac{\partial lnX_{CMC}}{\partial T}\right)$$

(4) $$\Delta S_{m}^{o}=\left(\Delta H_{m}^{o}-\Delta G_{m}^{o}\right)/T$$

The ln*X_cmc_* is dependent on temperature and can be described as by Equation (5) [62,63,64,65].

(5) $$lnX_{CMC}=A+BT+CT^{2}$$

**Figure 2:**
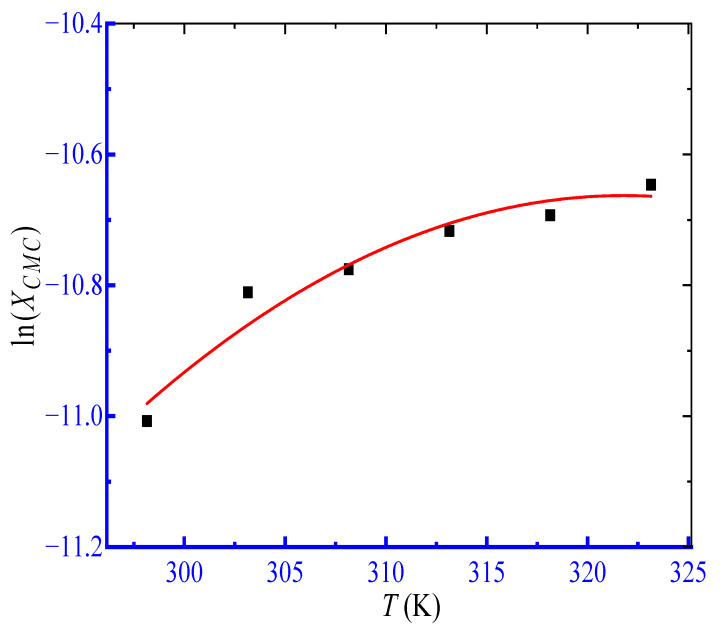
A second-order polynomial fitting of $lnX_{CMC}$ vs. *T* plot for the assembly of CPC + 0.1% (*w*/*v*) PVP systems in water.

A second-order polynomial fitting of $lnX_{CMC}$ vs. *T* plot was achieved nonlinear (Figure 2). The values of fitting parameters (*A*, *B*, and *C* (regression constants)) have been exposed in Table 5. The enthalpy ($\Delta H_{m}^{o}$) has thus been computed applying the following Equation (6):

(6) $$\Delta H_{m}^{o}=-\left(1+\beta \right)RT^{2}\left(B+2CT\right)$$

**Figure 3:**
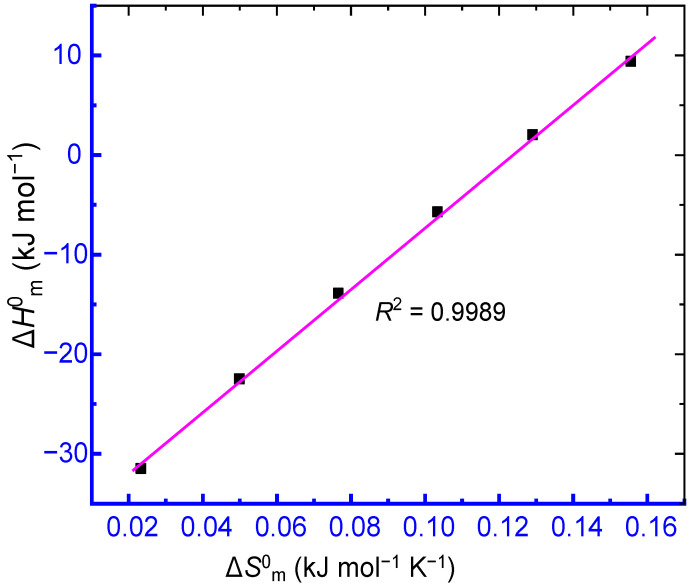
Enthalpy change vs. entropy change plot for the aggregation of CPC + 0.1% (*w*/*v*) PVP mixed system in water.

The $\Delta H_{m}^{o}$ and $\Delta S_{m}^{o}$ values achieved in the current study are not exposed in the table, but these values are used to determine the $\Delta H_{m}^{o}$-$\Delta S_{m}^{o}$ compensation. The enthalpy–entropy compensation (Figure 3) has been calculated from a linear connection amid $\Delta H_{m}^{0}$ and $\Delta S_{m}^{0}\;$with *R*^2^ value in the range of 0.9989–0.9992 using the following equation [66,67,68,69,70,71,72,73,74,75]:

(7) $$\Delta H_{m}^{0}=\Delta H_{m}^{0,*}+Tc_{\;}\Delta S_{m}^{0}$$

The compensation temperature, *T*_c_, and the intrinsic enthalpy gain, $\Delta H_{m}^{0,*}$ are represented by the slope and intercept, respectively. Table 6 shows the values of $\Delta H_{m}^{0,*}$ and *T*_c_ for the CPC + 0.1% (*w*/*v*) PVP systems in H_2_O and aq. salts solution.

According to Equation (7) above, the *R*^2^ value in the range of 0.9989–0.9992 was given in Table 6. The *T*_c_ and $\Delta H_{m}^{0,*}$describe the solute–solute and solute–solvent interactions, respectively, for the self-assembly process of amphiphiles [75]. The greater negative $\Delta H_{m}^{0,*}$ value indicates that micellization is preferred even when $\Delta S_{m}^{0}$ = 0 [70,71]. If entropy change value becomes zero, $\Delta H_{m}^{o}$ becomes equal to $\Delta H_{m}^{0,*}$ which refer the solute-solute interactions and the contribution of solvent effect might be ignored [73]. *T*_c_ values in this study were achieved in the range of 305.62–324.9 K. Shi et al. reported the *T*_c_ values of 312–321 K for the micellization of anionic/cationic/zwitterionic/nonionic amphiphiles. Koya et al. achieved the *T*_c_ and $\Delta H_{m}^{0,*}$ values of 220 K and −36.6 kJ mol^−1^ for the micellization of CPC in 0.1 mol L^−1^ glycine solution [74]. With a few exceptions, the *T*_c_ values for CPC + PVP were found to be nearly comparable to biological fluid [72]. Sugihara and Hisatomi [70] discovered a similar compensatory effect for the aggregation of charged amphiphiles in H_2_O medium. The *T*_c_ values have been described as the proof of hydrophobic interaction between the studied components [71]. García-Mateos et al. suggested the presence of hydrophobic interactions between CPC and PVP [31]. On the basis of higher negative $\Delta H_{m}^{0,*}$ and greater *T*_c_ values, Shi et al. reported that zwitterionic surfactants form more tighter and stable micelles compared to the anionic/cationic and nonionic amphiphiles [73].

## 4. Conclusions

The conductivities of PVP + CPC mixed systems were measured in H_2_O/H_2_O + NaCl/H_2_O + Na_2_SO_4_ solutions at various temperatures to insight into the interaction between PVP and CPC. The degree of interaction was determined by the values of *CMC*, *β*, and certain thermodynamic factors. Both the micellization of PVP + CPC mixed systems and *β* values were temperature dependent in aqueous and electrolytes media. At all temperatures, the values of $\Delta H_{m}^{o}$ and $\Delta S_{m}^{o}$ indicate the presence of hydrophobic interaction between PVP and CPC in aqueous and electrolytes media. The Δ*G^0^*_1,m_ values show that, the spontaneity of self-aggregation is almost similar in case of water and aq. NaCl medium whereas the negative values are lower in aq. Na_2_SO_4_ solutions. The values of $\Delta H_{m}^{o,*}$ vary from −35.66 to −38.05 kJ.mol^−1^, indicating that the micelle produced is stable. The *T*_c_ values are very similar to those of a biological system. Here, investigation of the interaction of surfactant and polymer in presence of low molecular weight electrolyte was carried out because low to moderate amphiphiles concentrations are used to form hydrogels, as self-assembly makes several approaches to attain gelation available.

## Figures and Tables

**Table 1:** Information of used materials.

Name of the Materials	Sources	Purity in Mass Fraction	CAS Number	Molecular Weight (g/mol)
CPC	Sigma-Aldrich, st. louis, MAUSA	0.99	124-03-8	340
PVP	Sigma-Aldrich, st. louis, MAUSA	0.99	9003-39-8	360,000
NaCl	Merck, Gurgaon-12, India	0.99	7647-14-5	58.44
Na_2_SO_4_	Merck, Mumbai, India	0.99	7757-82-6	142.04

**Table 2:** The *CMC* values of CPC + PVP assembly in H_2_O having different concentrations of PVP at 303.15 K.

*c*_PVP_	*CMC*_1_	*CMC*_2_	*CMC*_3_
% (*w*/*v*)	mmol kg^−1^	mmol kg^−1^	mmol kg^−1^
0.01	0.67	2.27	6.06
0.03	0.74	2.68	6.22
0.05	0.83	2.88	6.47
0.10	1.12	3.49	7.44

**Table 3:** The *CMC* values of the CPC + 0.1% (*w*/*v*) PVP mixture in H_2_O, H_2_O + NaCl, and H_2_O + Na_2_SO_4_ media at different temperatures.

Media	*I*_salts_	*T*	*CMC*_1_	*CMC*_2_	*CM*_3_
	mmol kg^−1^	K	mmol kg^−1^	mmol kg^−1^	mmol kg^−1^
H_2_O	0	298.15	0.92	3.03	7.31
		303.15	1.12	3.49	7.44
		308.15	1.16	3.71	7.71
		313.15	1.23	3.95	7.81
		318.15	1.26	3.82	7.62
		323.15	1.32	3.32	7.13
H_2_O + NaCl	1.50	298.15	0.78	4.29	8.01
		303.15	0.73	4.06	7.73
		308.15	0.57	3.15	7.66
		313.15	0.71	2.97	7.43
		318.15	0.77	2.49	7.45
		323.15	0.83	2.36	7.54
H_2_O + Na_2_SO_4_	1.50	298.15	0.43	4.65	8.51
		303.15	0.55	5.31	8.22
		308.15	0.51	4.81	8.11
		313.15	0.48	4.47	8.48
		318.15	0.44	4.27	9.02
		323.15	0.39	3.98	9.09

**Table 4:** Values of *β*_1_, free energy change (Δ*G*^0^_1,*m*_), and free energy change of transfer (Δ*G*^0^_1,*t*_) for CPC + 0.1% (*w*/*v*) PVP mixed systems in H_2_O and aq. solution of NaCl/Na_2_SO_4_.

Media	*I*_salts_	*T*	*X*_CMC_ (×10^5^)	*β*_1_	Δ*G^0^*_1,m_	Δ*G^0^*_1,t_
	mmol kg^−1^	K			kJ mol^−1^	kJ mol^−1^
H_2_O	0	298.15	1.658	0.67	−36.99	
		303.15	2.018	0.65	−37.08	
		308.15	2.090	0.65	−37.54	
		313.15	2.216	0.64	−37.87	
		318.15	2.270	0.63	−38.34	
		323.15	2.379	0.62	−39.00	
H_2_O + NaCl	1.50	298.15	1.406	0.69	−37.05	−0.0599
		303.15	1.315	0.68	−37.60	−0.5124
		308.15	1.027	0.68	−38.26	−0.7106
		313.15	1.279	0.67	−38.78	−0.9095
		318.15	1.388	0.65	−38.91	−0.5689
		323.15	1.496	0.64	−39.23	−0.2351
H_2_O + Na_2_SO_4_	1.50	298.15	0.775	0.35	−29.39	7.596
		303.15	0.991	0.34	−29.78	7.304
		308.15	0.919	0.39	−31.45	6.096
		313.15	0.865	0.50	−34.31	3.554
		318.15	0.793	0.51	−34.85	3.494
		323.15	0.703	0.53	−35.83	3.165

**Table 5:** The values of *A*, *B*, and *C* for the aggregation of CPC + 0.1% (*w*/*v*) PVP mixed system.

Media	I_salts_ mmol kg^−1^	*A*	*B*	*C*
H_2_O	0	−69.463	0.3654	−0.0006
H_2_O + NaCl	1.5	126.7	−0.8927	0.0014
H_2_O + Na_2_SO_4_	1.5	−139.01	0.8273	−0.0013

**Table 6:** Enthalpy–entropy compensation parameters for CPC + 0.1% (*w*/*v*) PVP systems in H_2_O and aq. salts solution.

Media	*I*_salts_	$\Delta H_{m}^{0,*}$	*T*_c_	*R* ^2^
	mmol kg^−1^	kJ mol^−1^	(K)	
H_2_O	0	−38.05	309.06	0.9989
H_2_O + NaCl	1.50	−37.32	305.62	0.9992
H_2_O + Na_2_SO_4_	1.50	−35.66	324.90	0.9991
